# Factors associated with mortality after snakebite envenoming in children: a scoping review

**DOI:** 10.1093/trstmh/trad031

**Published:** 2023-06-02

**Authors:** Lucy Guile, Adrienne Lee, José María Gutiérrez

**Affiliations:** Peninsula Medical School, University of Plymouth, Plymouth PL6 8BU, UK; Department of Anaesthesia, University Hospitals Plymouth NHS Trust, Plymouth PL6 8DH, UK; Peninsula Medical School, University of Plymouth, Plymouth PL6 8BU, UK; Department of Anaesthesia, University Hospitals Plymouth NHS Trust, Plymouth PL6 8DH, UK; Instituto Clodomiro Picado, School of Microbiology, University of Costa Rica, San José 11501, Costa Rica

**Keywords:** child mortality, envenoming, mortality, neglected tropical diseases, snakebite, snakebites

## Abstract

Snakebite envenoming is an important public health issue in many tropical and subtropical countries, where the burden of morbidity and mortality falls particularly on impoverished rural communities. Children are an especially vulnerable group. This scoping review provides an overview of the extent, type and content of peer-reviewed evidence regarding factors associated with mortality in snakebite-envenomed children. A comprehensive literature search of MEDLINE and the Global Index Medicus yielded 623 articles, of which 15 met the criteria for inclusion; 67% of studies were conducted in India, with the remaining studies taking place in Papua New Guinea, Morocco and The Gambia. There was a notable scarcity of eligible studies from sub-Saharan Africa and Latin America despite the high burden of envenoming in these regions. The risk factors for mortality that were identified by the greatest number of studies were younger patient age (n=4), delay in administration of antivenom (n=4) and acute kidney injury (n=3). Identification of poor prognostic factors can assist clinicians in making timely referrals to centres with paediatric critical care capability. Future research must address the lack of studies from key geographical regions so that evidence-based improvements to the care of this vulnerable group can be implemented.

## Introduction

Snakebite envenoming is a major cause of death and disability in Asia, Africa, Latin America and parts of Oceania. An estimated 63 400^1^ to 138 000 deaths^2^ occur each year as a consequence of these envenomings, and an even higher number of people are left with permanent physical and psychological sequelae.^3^ Underreporting of snakebite and its resulting morbidity and mortality is widespread in many regions, for reasons that include lack of coordinated data collection and the fact that not all cases present to health services.^4,5^

Owing to the large variation in snake venom composition and mechanisms of action, the clinical manifestations of envenomings vary depending on the species. Neurotoxic manifestations predominate in envenomings by most species of the family Elapidae, although some elapids induce local necrosis. Snakes of the family Viperidae inflict envenomings characterised by local tissue damage, bleeding, coagulopathies, haemodynamic alterations and acute kidney injury.^3,6^ The mainstay of therapy for snakebite envenoming is the administration of animal-derived antivenoms.^7^

Snakebite envenoming was formally recognised as a neglected tropical disease by the WHO in 2017. A resolution on snakebite envenoming was passed by the World Health Assembly in 2018, and a WHO global strategy for prevention and control was launched in 2019.^8,9^ Children are particularly susceptible to complications of envenoming, which may be as a result of factors such as a lower volume of distribution due to smaller body size.^10^ Children in low-income rural communities are especially vulnerable.^11^

Despite the magnitude of this problem, no comprehensive overview of risk factors and predictors of mortality after snakebite in children has been published. Existing studies that identify these factors are heterogenous, encompassing different patient populations, geographical regions, snake species, healthcare systems and study designs. To reduce the burden of mortality, in keeping with the WHO strategy for the prevention and control of snakebite envenoming,^9^ it is necessary to explore specific features that characterise the impact of this neglected tropical disease in the paediatric population.

The aim of this scoping review is to identify and map the extent, type and content of peer-reviewed evidence regarding risk factors and predictors of mortality after snakebite envenoming in children.

## Methods

This study aims to address the following two questions:

1. What factors are associated with mortality in snakebite-envenomed children?
2. What questions should be addressed by future research on this topic?

### Search strategy

The study protocol was informed by the Joanna Briggs Institute's methodology for scoping reviews^12^ and prospectively registered via the Open Science Framework Registry.^13^ The Preferred Reporting Items for Systematic reviews and Meta-Analyses extension for Scoping Reviews checklist was also utilised.^14^

An initial limited search of MEDLINE (via PubMed) was undertaken to identify articles on the topic. The text words contained within the titles and abstracts of relevant articles and the associated index terms were used to develop a full search strategy. The search terms and strategy were selected in discussion with a medical information specialist, using the Population, Exposure, Outcome framework. Piloting on a limited number of samples was conducted to ensure consistency in reviewer decision-making prior to undertaking a review of all articles.

A systematic search was conducted on 25 February 2023 using MEDLINE (via PubMed) and the Global Index Medicus. The Global Index Medicus encompasses five regional databases of health-related literature produced by and within low- to middle-income countries. The search was performed in English, Spanish and Portuguese in all databases. A hand search of the reference lists of included articles was performed to identify additional articles.

Full electronic search strategies are available in Supplementary File S1.

### Eligibility criteria

Included were observational or interventional studies that reported factors associated with mortality after snakebite envenoming in children, with associated numerical estimate of relative risk, odds ratio or similar.

There was no restriction by date of publication, geographical location or language of publication. Studies were excluded if: no patients died; children were grouped or analysed with individuals aged ≥18 y; there was no quantitative estimate of risk associated with the proposed factor/s with associated assessment of statistical significance.

### Data extraction

Article inclusion and exclusion were determined by two reviewers working independently. LG and AL or LG and JMG initially reviewed each abstract and/or full text. Discrepancies were resolved by consensus or discussion with the third reviewer. Data were extracted to Microsoft Excel (v. 2210, Microsoft Corp., Redmond, WA, USA) using the piloted spreadsheet (Supplementary File S2).

Key information from the included studies was summarised descriptively. Factors that were associated with mortality were categorised as ‘patient factors’, ‘treatment factors’ or ‘clinical features’. The heterogeneity of the studies precluded synthesis and inferential statistical analysis of compiled results. This is typical for scoping reviews, which aim to provide an overview of existing evidence rather than a critically appraised synthesis of results.^12,15^ Formal critical appraisal of the sources of evidence was not performed.

## Results

**Figure 1:**
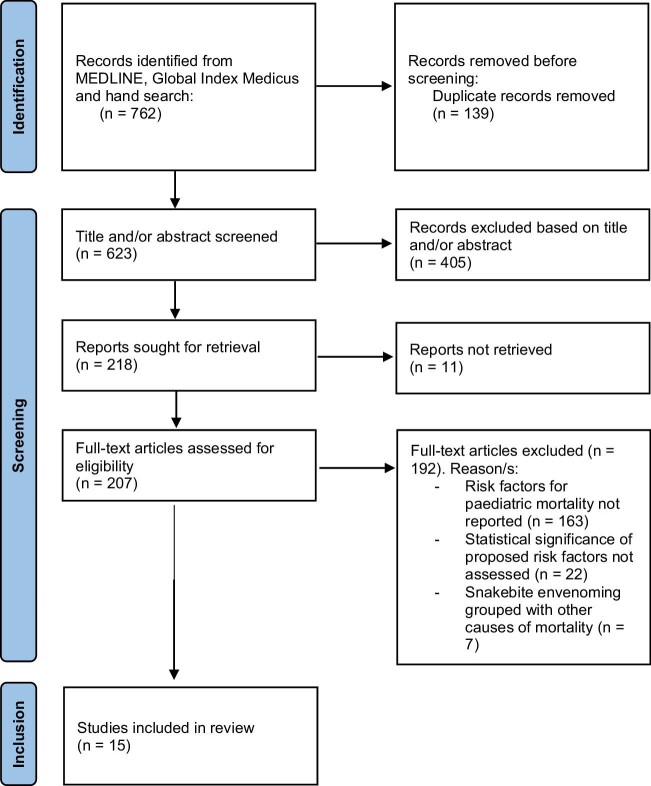
Inclusion decision flowchart.

In total, 623 articles were retrieved by the search strategy after removal of duplicates (Figure 1); 405 were excluded based on the title and/or abstract. Of the remaining 218, 11 articles could not be retrieved, so 207 full-text articles were assessed for eligibility; 192 of these were excluded, resulting in 15 included articles.

### Characteristics of included sources of evidence

The included studies were published from 1987 to 2022. The majority of studies (n=10; 67%) were conducted in India within the last 10 y (Table 1). Two studies (13%) were conducted in Papua New Guinea, two in Morocco and one in The Gambia.

**Table 1.** Overview of included studies and predictors of mortality

						Predictors of mortality that met statistical significance			
Authors (year of publication)	Study type	Study location	Sample size	Selected patient inclusion criteria	Study case fatality rate % (n/total)	Patient factors	Treatment factors	Clinical features	Comments
Essafti et al. (2022)^28^	R, O	Morocco	75	Admitted to PICU	8 (6/75)			AKI	
Hooda et al. (2021)^29^	R, O	India	68	Received AV	16 (11/68)		Severe reaction to AV		
Suryanarayana et al. (2021)^16^	R, O	India	308		12 (36/308)	Age ≤5 y Walking at the time of snakebite Playing at the time of snakebite	No tourniquet used Bite to AV time >6 h	Presence of fang marks Neurotoxic envenomation Requirement for repeated AV dose	Outcome analysed was mortality, need for mechanical ventilation or need for renal replacement therapy
Giri et al. (2020)^17^	P, O	India	47	Admitted to PICU	13 (6/47)		Bite to AV time >6 h		
Islam et al. (2020)^24^	R, O	India	364	Haemotoxic envenomation	4.4 (16/364)		Bite to AV time >1 h	Bleeding Requirement for mechanical ventilation	
Samprathi et al. (2020)^18^	R, O	India	51	Admitted to PICU with EMNS	14 (7/51)	Younger age^a^	Non-availability of intensive care beds^a^	Ptosis^a,b^ In cardiac arrest at admission^a^	
Shekar et al. (2020)^30^	P, I	India	62	Required mechanical ventilation due to paralysis	5 (3/62)				No mortality difference found between low- vs high-dose AV administration
Jayakrishnan et al. (2017)^19^	P, O	India	145	Admitted to PICU	10.3 (15/145)			AKI Severe leucocytosis on day 1 Capillary leak syndrome^a^ Nocturnal bites^a^ Requirement for >20 vials of AV^a^ Thrombocytopenia on day 1^a^	
El Hattimy et al. (2016)^25^	R, O	Morocco	265	Reported to national poison control centre (Le Centre Antipoison et de Pharmacovigil-ance du Maroc)	5.7 (15/265)	Age 5–9 y			
Krishnamurthy et al. (2015)^26^	P, O	India	61	Russell's viper envenomation	7 (4/61)			AKI	
Sankar et al. (2013)^20^	P, O	India	110		12.7 (14/110)	Age ≤6 y Walking for >1 km after the bite		Vomiting^a^ Haemoglobin ≤10 g/dl at admission Species of snake (cobra)	Outcome analysed was mortality or major disability
Waikhom et al. (2013)^21^	P/R, O	India	61	AKI due to Russell's viper envenomation	30 (18/61)		Longer time to AV administration	Bite during the winter season Hypotension at presentation	
McGain et al. (2004)^27^	R, O	Papua New Guinea	260	Admitted to ICU	14.6 (38/260)		Shorter median invasive ventilation time		
Enwere et al. (2000)^22^	R, O	The Gambia	28		14 (4/28)			Shock Adenitis Restlessness	
Brian, Vince (1987)^23^	R, O	Papua New Guinea	52	Admitted to ICU	8 (4/52)	Age ≤5 y		Requirement for intubation in <5-y-olds	

Abbreviations: AKI, acute kidney injury; AV, antivenom; EMNS, early morning neuroparalytic syndrome; I, interventional; ICU, intensive care unit; O, observational; P, prospective; PICU, paediatric intensive care unit; R, retrospective.

a Reported to be significant on univariable but not multivariable analysis.

b The reporting of this feature is ambiguous, with the text stating that presence of ptosis is associated with mortality but the table indicating that it is absence of ptosis that is significantly associated with mortality.

Fourteen included articles (93%) reported single-centre studies undertaken in secondary or tertiary care facilities, with sample sizes ranging from 28 to 364 patients (Table 1). One article reported results from a national database. Fourteen articles were published in English and one was published in French. Identification of risk factors for mortality after snakebite envenoming in paediatric patients was a primary aim in 53% of studies (8/15).^16–23^ Specified upper age limits for the patients included in the studies ranged from 11^18^ to 16 y^23^ (inclusive).

### Predictors of mortality

Paediatric case fatality rates within the studies ranged from 4.4% (16/364)^24^ to 30% (18/61).^21^ Predictors of mortality in the studies analysed are described in Table 1.

A range of statistical tests was used to identify factors that predict mortality. All studies quoted p values for each factor (Supplementary File S3). p≤0.05 was stated to indicate significance by 11 studies. The remaining four studies did not explicitly specify a threshold, but all the variables that were deemed significant did have p≤0.05.^22,25–27^

### Species of snake responsible for envenoming

Overall, 53% of studies (8/15) reported the snake species responsible for envenoming where this had been recorded (Table 2). This includes two studies that intentionally recruited only patients who were known or suspected to have been bitten by a particular species of snake. With respect to the four studies in which snakes were identified by being brought to hospital, it was not specified whether the identifications were made by medical personnel or by a herpetologist.

**Table 2.** Characteristics of snakes responsible for envenoming and of antivenom administered, by study

Authors (year of publication). Study location	Snake species responsible for envenoming	% (n/total) of patients in the study envenomed by each species	Method of identifying snake species	Type of antivenom administered (polyvalent/monovalent; manufacturer)	Number of vials of AV administered
Essafti et al. (2022).^28^ Morocco.	Lataste's viper (*Vipera latastei*) White-bellied carpet viper (*Echis leucogaster*) Saharan horned viper (*Cerastes cerastes*) Unidentified	8 (6/75) 5 (4/75) 3 (2/75) 84 (63/75)	Identified by patient using images	Polyvalent; FAV-Afrique; Sanofi Pasteur, Lyon, France (2013–2015) Inoserp-Mena; Inosan Biopharma, Mexico (2016–2020)	Actual doses administered not reported
Hooda et al. (2021).^29^ India	Not specified	ND	N/a	Polyvalent; Vins Bioproducts Ltd, Hyderabad, Telangana, India (2012–2015) Premium Serums and Vaccines Pvt. Ltd, Pune, Maharashtra, India (2015–2017)	15.8±6.7 Mean±SD
Suryanarayana et al. (2021).^16^ India	Russell's viper *(Daboia russelii)* Indian cobra (*Naja naja*) Common krait (*Bungarus caeruleus*) Saw-scaled viper (*Echis carinatus*) Unidentified	27.6 (85/308) 13.0 (40/308) 8.8 (27/308) 7.1 (22/308) 43.5 (134/308)	Snake brought to hospital OR Identified by patient/witness (not specified how)	Not specified	15 (10–20) Median (IQR)
Giri et al. (2020).^17^ India	Not specified	ND	N/a	Not specified	N/a
Islam et al. (2020).^24^ India	Not specified	ND	N/a	Not specified	N/a
Samprathi et al. (2020).^18^ India	Not specified	ND	N/a	Not specified	N/a
Shekar et al. (2020).^30^ India	Krait* Cobra* Unidentified	10 (6/62) 5 (3/62) 85 (53/62)	Snake brought to hospital	Polyvalent; Haffkine Institute, Mumbai, Maharashtra, India	Low dose group–10 High dose group–20
Jayakrishnan et al. (2017).^19^ India	Russell's viper (*Daboia russelii*) Hump-nosed pit viper (*Hypnale hypnale*) Common krait (*Bungarus caeruleus*) Indian cobra (*Naja naja*) Unidentified	22.1 (32/145) 10.3 (15/145) 1.4 (2/145) 1.4 (2/145) 64.8 (94/145)	Not specified	Polyvalent; not specified	N/a
El Hattimy et al. (2016).^25^ Morocco	Not specified	ND	N/a	Polvalent; FAV-Afrique; Sanofi Pasteur, Lyon, France	0–4 Range
Krishnamurthy et al. (2015).^26^ India	Russell's viper (*Daboia russelii*)	100 (61/61)	Snake brought to hospital OR Identified by patient/witness using images plus typical clinical presentation	Polyvalent; Bharat Serum and Vaccines Ltd, Mumbai, Maharashtra, India	10.9 (7.6) Mean (SD)
Sankar et al. (2013).^20^ India	Saw-scaled viper (*Echis carinatus*) Russell's viper *(Daboia russelii)* Common krait (*Bungarus caeruleus*) Indian cobra (*Naja naja*) Unidentified	38.2 (42/110) 14.5 (16/110) 10.0 (11/110) 9.1 (10/110) 26.4 (29/110)	Not specified	Polyvalent; Serum Institute of India, Pune, Maharashtra, India	15 (10–19) Median (IQR)
Waikhom et al. (2013).^21^ India	Russell's viper (*Daboia russelli*)	100 (61/61)	Snake brought to hospital OR Identified by patient/witness using images plus typical clinical presentation	Polyvalent; Bharat Serum and Vaccines Ltd, Mumbai, Maharashtra, India	Actual doses administered not reported
McGain et al. (2004).^27^ Papua New Guinea	Not specified	ND	N/a	Polyvalent Monovalent (taipan) Monovalent (death adder); CSL Limited, Melbourne, Victoria, Australia	0–2 Range
Enwere et al. (2000).^22^ Gambia	Not specified	ND	N/a	Polyvalent; not specified	N/a
Brian, Vince (1987).^23^ Papua New Guinea	Papuan black snake (*Pseudechis papuanus*) Unidentified	3.8 (2/52) 96.2 (50/52)	Not specified	Polyvalent Monovalent (species not specified); Commonwealth Serum Laboratories, Melbourne, Victoria, Australia	0–2 Range

Abbreviations: AV, antivenom; N/a, not applicable; ND, no data.

*exact species not recorded.

Where antivenom type was not specified, the number of vials was recorded in the table as ‘N/a’, as assessment of the adequacy of dosing is not possible without this data.

In total, 73% of studies (11/15) reported the administration of polyvalent antivenom to patients, two of which reported that some patients received polyvalent antivenom and others received monovalent antivenom. Details of the type of antivenom provided in the remaining four studies were not provided.

## Discussion

### Summary of evidence

This scoping review identifies a number of factors reported to be associated with mortality in paediatric victims of snakebite envenoming in India, Papua New Guinea, Morocco and The Gambia. The key patient characteristic that emerges as a risk factor for mortality is younger age, which was found by four studies. A fifth study found the 5–9 y age group at a higher risk of death after snake envenoming compared with both 10–14- and 1–4-y-olds.^25^ The study authors do not offer a hypothesis as to why 5–9-y-olds appear to be at a higher risk than the younger age group, which is an unexpected finding. Younger age as a risk factor is likely to be due to lower body mass and, consequently, a lower volume of distribution of venom. None of the studies assessed absolute body mass as a potential risk factor for severity of envenoming. One study considered severe malnutrition but did not find this to be a statistically significant risk factor for mortality or major disability.^20^ In addition to body mass, other as yet unknown physiological factors may affect younger children's susceptibility to envenoming. For example, the venom of *Daboia russelii* exerts a stronger in vitro procoagulant activity on paediatric plasma compared with adult plasma,^31^ suggesting that children may have a higher risk of developing coagulopathy after envenoming by this species.

The identification of rural residence and walking for more than one kilometre after envenoming as risk factors is consistent with the observation that impoverished rural communities carry the greatest burden of envenoming-related mortality.^8^ Several factors are likely to favour mortality in such settings, such as difficulty in accessing health services, and poor availability of antivenoms and other therapeutic devices (e.g. mechanical ventilators to manage severe neurotoxic envenoming) in health facilities.^32^ Limited medical and nursing personnel and non-adherence to therapeutic protocols for managing snakebite envenoming and its complications may also contribute.^33^ Delays in access to health services are particularly relevant in the case of envenomings that may rapidly evolve in severity, such as some neurotoxic envenomings.^32^

With respect to treatment factors, four studies found that a longer time from bite to administration of antivenom was associated with mortality. Delay in antivenom administration has been repeatedly associated with poor prognosis in envenoming of adults caused by different snake species in various settings.^34–37^ A severe adverse reaction to antivenom was identified as a risk factor for death by one study.^29^ A high incidence of adverse reactions to antivenoms can be related to poor physicochemical quality of products, because factors such as the presence of protein aggregates, non-immunoglobulin contaminating proteins or bacterial endotoxins are associated with high rates of adverse clinical manifestations.^38^ In addition, it is generally recommended that the dose of antivenom be the same in children and in adults,^39^ thus reaching higher concentration in the blood of children. Because some adverse reactions to antivenoms are dose-related, this might be another factor that contributes to complications following snakebites in children. Conversely, a review article by Oliveira et al. cites underdosing of antivenom as a risk factor for clinical severity following *Bothrops* envenoming in Brazilian children.^40^

Access to safe and effective antivenoms is fundamental for reducing snakebite envenoming morbidity and mortality worldwide.^41^ To be effective, antivenoms must be manufactured with reference to the taxonomic and venom proteomic profile of venomous snakes in the geographical area of operation and should be subject to rigorous preclinical assessment of efficacy and quality control processes.^42^ Other important logistical challenges to widespread availability of antivenoms include a robust cold chain (for liquid antivenoms), and effective health information systems to accurately assess the incidence and type of snake envenoming, to facilitate evidence-based intervention and distribution of resources.^42^ The infrastructure of some of the regions that experience the greatest burden of snakebite envenoming—such as sub-Saharan Africa and Asia—is frequently unable to meet these requirements.^43^

Resource limitation is cited as a key challenge in many of the studies identified by our scoping review. The authors of one of the two studies from Papua New Guinea comment that rates of both intubation and mortality for snakebite-envenomed children admitted to their study site were substantially higher in their case series (1991–2001) than in a previous study from the 1980s. They explicitly link this worsening of outcomes to diminished access to antivenom due to rising costs.^27^ Lack of ventilators in some centres means that hand ventilation by relatives can be required,^27^ and high patient-to-staff ratios can make adequate monitoring of intubated patients challenging. Problems with endotracheal tube patency and position were reported in all four children who died in another of the studies.^23^

Almost half of the studies (7/15) did not record the species of snakes responsible for envenoming (Table 2), although all discussed the species endemic to the study locality. This is a common issue in epidemiological studies of snakebite. Identification of the envenoming species can guide management, but the ability of patients and witnesses to reliably do so is often poor.^39^ Of the studies that did document the snake species, three did not specify how this identification was made. In other cases, the snake was brought to the hospital, or identified by patients or witnesses, which is not a highly reliable method. Two studies reported using clinical presentation to inform the identification of the envenoming species. Snake identification is relevant for epidemiological studies and for the characterisation of the clinical profile of envenomings. A syndromic approach to the diagnosis of envenoming is useful when the culprit snake is unknown.^44^ In the case of India, where most of the studies hereby reviewed were conducted, polyvalent antivenoms are generally used, which neutralise the venoms of the most relevant snakes in that country. Venom detection kits are sometimes used in Australia and Papua New Guinea for the identification of the envenoming species,^45^ but this diagnostic tool can produce erroneous results^46^ and is unavailable in other regions of the world.

A broad range of clinical features were identified as predictors of mortality. Acute kidney injury was identified by three studies, two of which were conducted in India and one of which was conducted in Morocco. This feature is highly prevalent in envenoming by *D. russelii*,^39^ which was responsible for many of the Indian cases analysed in this review. This constitutes a challenge because in many countries renal replacement therapy is not widely available, particularly for young children.^47^ However, because the incidence of acute kidney injury is higher in envenomings by *D. russelii* than in those inflicted by other species, this predictor cannot necessarily be generalised to envenomings by different species, and further studies need to be carried out to identify clinical features predictive of mortality in other types of envenomings. Hypotension was identified as a statistically significant risk factor by two studies and capillary leak syndrome and abnormal bleeding by one study each, reflecting cardiovascular and haematological compromise. Several other predictors relate to neurotoxic envenoming and highlight the importance of the availability of mechanical ventilation for severe cases, where respiratory paralysis is an important cause of death. Some factors—such as ‘bite during the winter season’—are difficult to explain and could result from confounding factors (such as differences in time to presentation at hospital as a result of seasonal changes in travelling conditions), or from methodological flaws in the studies.

### Limitations

This review did not formally assess the quality of the included studies. Although typical of scoping reviews, this limits the ability of the findings to be directly applied to clinical decision-making. Furthermore, with one exception, the studies that investigate association between treatment factors and mortality are all observational, meaning that causation cannot be confidently established.

Fourteen studies were hospital-based, the majority in tertiary referral centres, and the other included study relied upon hospital records submitted to a central database. Many snakebite envenoming victims die before reaching a healthcare facility.^5^ These deaths clearly cannot be captured by hospital-based studies.

It is noteworthy that all 14 of the included studies came from just four countries. Despite the fact that an estimated 7000–32 000 snakebite envenoming deaths occur annually in sub-Saharan Africa and 3400–5 000 in Latin America and the Caribbean,^3^ only one qualifying study from these regions was identified. One large Brazilian review article did not meet the inclusion criteria but aligned with the findings of our review in identifying younger age, rural residence, time from bite to medical care >3 h and unclottable blood as statistically significant risk factors for severity following *Bothrops* envenoming in children.^40^ However, as the majority of the studies included in this review are from India, the conclusions regarding risk factors for mortality cannot be directly extrapolated to envenoming caused by snakes predominant in other regions. Similarly, all but one of the included papers described single-centre studies. Although comprehensive, it is possible that our search strategy failed to identify grey literature that would have been relevant to our objectives. Overall, these limitations highlight the relative dearth of evidence about the clinical effects of snakebite in this particularly vulnerable group.

## Conclusions

This scoping review highlights younger age—particularly age 0–5 y—acute kidney injury and delay between bite and antivenom administration as factors frequently associated with mortality in snakebite-envenomed children in the contexts studied. Recognition of predictors of mortality can aid in the timely referral of patients to specialist centres with paediatric critical care capability. However, even within paediatric critical care environments, resource limitation such as lack of access to antivenom, mechanical ventilation or renal replacement therapy can put patients at risk of poor outcomes. The reasons for delay between snakebite occurrence and administration of antivenom are multifactorial and specific to different localities, with influence from geographical, logistical and sociocultural factors. Multidisciplinary collaboration involving social scientists may aid the understanding of these delays in many settings.^48^

This scoping review clearly identifies the need for high quality research that examines factors associated with paediatric mortality after snakebite envenoming in a broader range of geographical regions, including sub-Saharan Africa, Latin America and Asian countries other than India. Prospective multicentre studies would provide a firmer evidence base for the care of this vulnerable group of patients. Because the pathophysiological features of envenoming vary between different snake species, it is necessary to assess how different clinical profiles of envenoming affect children in settings with variable cultural, ecological and public health features. This requires studies to have robust methods of identifying envenoming species. Likewise, the identification of comorbidities that may impact on the severity of envenoming in children is another issue that deserves attention. Identification of these factors will enable the evidence-based targeting of resources to minimise both morbidity and mortality in the many children globally who are affected by snake envenoming.

## Supplementary Material

trad031_Supplemental_Files

## Data Availability

The data supporting the findings of this article are available within the article and its supplementary materials.
