# Supplementary material for: Factors associated with mortality after snakebite envenoming in children: a scoping review
Source: Trans R Soc Trop Med Hyg. 2023 Jun 2;117(9):617–27. doi: 10.1093/trstmh/trad031 (PMC10472879; doi:10.1093/trstmh/trad031)
Supplement: trad031_Supplemental_Files [file trad031_supplemental_files.zip › SUPPLE~1.DOC]

**Supplementary File S3. Table of statistical tests and associated values**

| **Authors. Year of publication** | **Predictors of mortality that met statistical significance** | **Statistical test used** | **Value of statistical test** |
| --- | --- | --- | --- |
| Essafti et al. (2022)^28^ | AKI (acute kidney injury) | Univariate and bivariate analyses | p = 0.04 |
| Hooda et al. (2021)^29^ | Severe reaction to AV | Chi-square | p = 0.005 |
| Suryanarayana et al. (2021)^16^ | Age ≤5 years  Walking at the time of snakebite  Playing at the time of snakebite  No tourniquet use  Bite to AV time more than six hours  Presence of fang marks  Neurotoxic envenomation  Requirement for repeated AV dose | Adjusted odds ratio (95% confidence interval, p-value) | 2.97 (1.28-6.90, 0.012)  6.15 (2.88-13.17, <0.001)  3.36 (1.64-6.88, 0.001)  2.39 (1.125-4.57, 0.008)  2.71 (1.45-5.06, 0.002)  2.22 (1.21-4.07, 0.01)  3.01 (1.11-8.13, 0.03)  8.41 (2.99-23.60, <0.001) |
| Giri et al. (2020)^17^ | Bite to AV time more than six hours | Chi-square | p <0.05 |
| Islam et al. (2020)^24^ | Bite to AV time more than one hour  Bleeding  Requirement for mechanical ventilation | Adjusted odds ratio (95% confidence interval, p-value) | 15.2 (14.7-15.7, <0.05)  9.5 (8.5-10.6, <0.05)  13.8 (12.6-15.0, <0.05) |
| Samprathi et al. (2020)^18^ | Younger age^a^  Nonavailability of intensive care beds^a^  Ptosis^a,b^  In cardiac arrest at admission^a^ | Mann-Whitney U test for continuous variables,  Fisher’s exact test for dichotomous outcomes | p = 0.013  p = 0.005  p = 0.031  p= 0.002 |
| Shekar et al. (2020)^30^ | Nil - no difference found between low versus high-dose AV administration | Chi-square | p-value NS |
| Jayakrishnan et al. (2017)^19^ | Nocturnal bites^a^  Severe leucocytosis on day 1  Thrombocytopenia on day 1  AKI  Capillary leak syndrome^a^  Requirement for more than 20 vials of AV^a^ | Risk ratio (95% confidence interval, p-value) | 3.03 (1.01-9.07, 0.04)  18.28 (7.97-41.91, <0.001)  14.04 (5.86-33.63) <0.001  12.11 (3.62-40.52, <0.001)  27.00 (11.42-63.81, <0.001)  12.36 (3.77-40.54, <0.001) |
| El Hattimy et al. (2016)^25^ | Age 5-9 years | Chi-square, risk ratio (95% confidence interval, p-value) | 4.11, 3.37 (1.15-9.8, 0.04) |
| Krishnamurthy et al. (2015)^26^ | AKI | Chi-square or Fisher’s exact test | p = 0.039 |
| Sankar et al. (2013)^20^ | Age ≤6 years  Walking for >1 km after the bite  Vomiting^a^  Haemoglobin ≤10 g/dl at admission Species of snake (cobra) | Odds ratio (95% confidence interval, p-value) | 1.82 (1.17-2.83, 0.01)  18.4 (2.32-146.4, 0.001)  1.41 (1.12-1.79, 0.02)  3.62 (2.24-5.84, <0.001)  8.7 (1.3-57, 0.02) |
| Waikhom et al. (2013)^21^ | Longer time to AV administration  Bite during the winter season  Hypotension at presentation | Pearson correlation coefficient (r, p-value) | 0.565, <0.01  −0.459, <0.01  0.676, <0.01 |
| McGain et al. (2004)^27^ | Shorter median ventilation time (days) | Student’s t test - mean (p-value) | 3.0 versus 4.5 (<0.02) |
| Enwere et al (2000)^22^ | Shock  Adenitis  Restlessness | Chi-square | p < 0.005  p < 0.005  p < 0.05 |
| Brian, Vince (1987)^23^ | Age ≤5 years  Requirement for intubation in under 5 year-olds | Chi-square | p = 0.05  p = 0.003 |

AKI = acute kidney injury; AV = antivenom; EMNS = early morning neuroparalytic syndrome; I = interventional; ICU = intensive care unit; ND = no data; NS = not significant (p >0.05); O = observational; P = prospective; PICU = paediatric intensive care unit; R = retrospective.

^a^ Reported to be significant on univariable but not multivariable analysis

^b^ The reporting of this feature is ambiguous, with the text stating that presence of ptosis is associated with mortality but the table indicating that it is absence of ptosis that is significantly associated with mortality
