# Supplementary material for: Factors associated with mortality after snakebite envenoming in children: a scoping review
Source: Trans R Soc Trop Med Hyg. 2023 Jun 2;117(9):617–27. doi: 10.1093/trstmh/trad031 (PMC10472879; doi:10.1093/trstmh/trad031)
Supplement: trad031_Supplemental_Files [file trad031_supplemental_files.zip › SUPPLE~2.DOC]

**Supplementary File S1. Electronic search strategies**

MEDLINE (via Pubmed) - English:

((((((((child*) OR (infant*)) OR (baby)) OR (neonate*)) OR (paediatric)) OR (pediatric)) AND (("Snake Bites"[Mesh]) OR ((((snake envenoming*) OR (snake bite*)) OR (snakebite*)) OR (snake envenomation*)))) AND (((mortality) OR (death*)) OR (((("Mortality"[Mesh]) OR "Child Mortality"[Mesh]) OR "Infant Mortality"[Mesh]) OR "Infant Death"[Mesh])))

MEDLINE (via Pubmed) – Spanish:

(((niño*) OR (niña*) OR (bebé*) OR (pediátr*)) AND ((envenenamento* por serpente*) OR (mordida* de serpente*) OR (picada* de cobra*) OR (mordedura* de víbora*))) AND ((mortalidad) OR (muerte*) OR (letalidad))

MEDLINE (via Pubmed) – Portuguese:

(((criança*) OR (filh*) OR (bebê*) OR (pediátr*)) AND ((envenenamento* por serpente*) OR (mordida* de serpente*) OR (picada* de cobra*))) AND ((mortalidade) OR (morte*) OR (óbito*))

Globus Index Medicus – English:

(tw:((child*) OR (infant*) OR (baby) OR (neonate*) OR (paediatric) OR (pediatric))) AND (tw:((snake envenoming) OR (snake bite*) OR (snakebite*) OR (snake envenomation*) OR ( mh:("Snake Bites")))) AND (tw:((mortality) OR (death*) OR ( mh:("Child Mortality")) OR ( mh:("Infant Mortality")) OR ( mh:("Mortality")) OR ( mh:("Infant Death"))))

Globus Index Medicus – Spanish:

(tw:((niño*) OR (niña*) OR (bebé*) OR (pediátr*))) AND (tw:((envenenamento* por serpente*) OR (mordida* de serpente*) OR (picada* de cobra*) OR (mordedura* de víbora*))) AND (tw:((mortalidad) OR (muerte*) OR (letalidad)))

Global Index Medicus – Portuguese:

(tw:((criança*) OR (filh*) OR (bebê*) OR (pediátr*))) AND (tw:((envenenamento* por serpente*) OR (mordida* de serpente*) OR (acidentes ofídicos) OR (ofidismo) OR (picada* de cobra*))) AND (tw:((mortalidade) OR (morte*) OR (óbito*)))
